# Severe, Recurrent Crusted Scabies in a Psoriatic on Methotrexate

**DOI:** 10.4269/ajtmh.22-0008

**Published:** 2022-03-21

**Authors:** Ananta Khurana, Savitha B, Aishwarya Muddebihal, Arvind Ahuja

**Affiliations:** ^1^ Department of Dermatology, Atal Bihari Vajpayee Institute of Medical Sciences and Dr Ram Manohar Lohia Hospital, New Delhi, India;; ^2^ Department of Pathology, Atal Bihari Vajpayee Institute of Medical Sciences and Dr Ram Manohar Lohia Hospital, New Delhi, India

Crusted scabies results from hyperinfestation with *Sarcoptes scabiei* and is believed to be consequent to certain predisposing factors such as immunosuppression, systemic lupus erythematosus, Down syndrome, immunodeficiency disorders, neurological disorders, and more, rather than by progression from ordinary scabies.

A 23-year-old woman, a known case of psoriasis vulgaris with an unstable disease course, presented twice within a span of 6 months with crusted scabies localized to acral parts. Both these episodes occurred while she was taking methotrexate (7.5 mg/week and 10mg/week, respectively). On both occasions, the patient was treated with two weekly doses of ivermectin, to which she responded completely, and methotrexate was continued.

**Figure 1.**
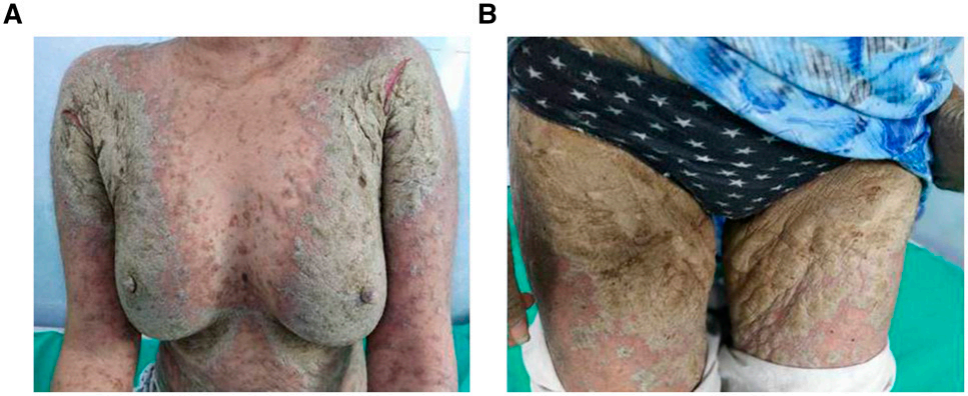
(**A, B**) Extensive crusted scabies with thick, grayish yellow crusting, especially around the limb girdles. Linear fissures are also visible in the axillary regions (**A**). No defined psoriatic plaques could be made out at this time. This figure appears in color at www.ajtmh.org.

**Figure 2.**
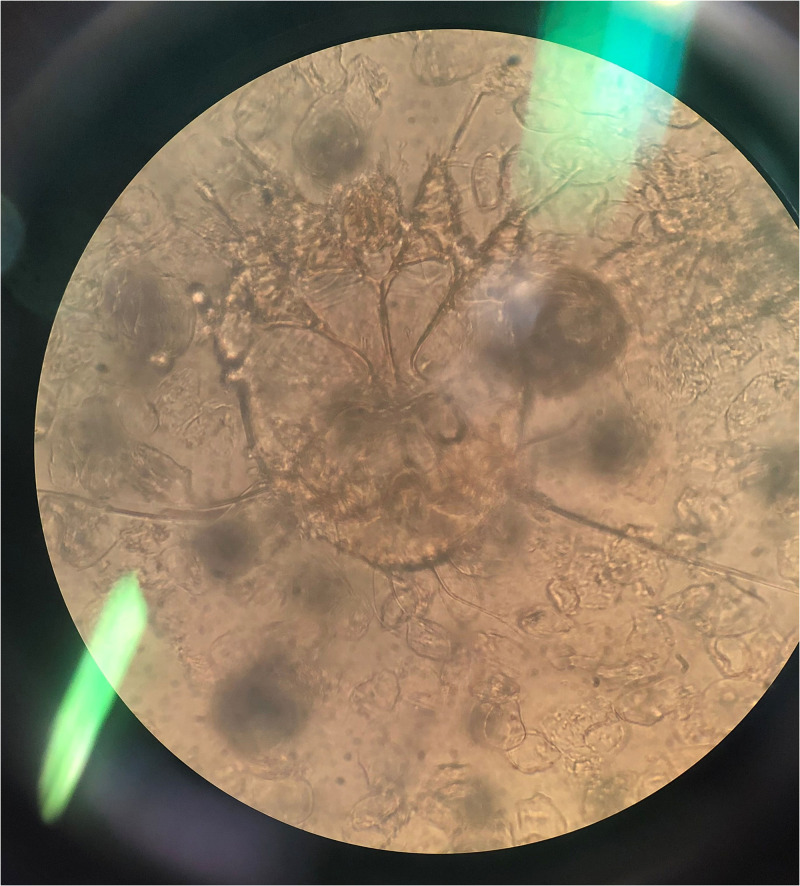
Scabies mite in a smear made from crusted lesions. This figure appears in color at www.ajtmh.org.

**Figure 3.**
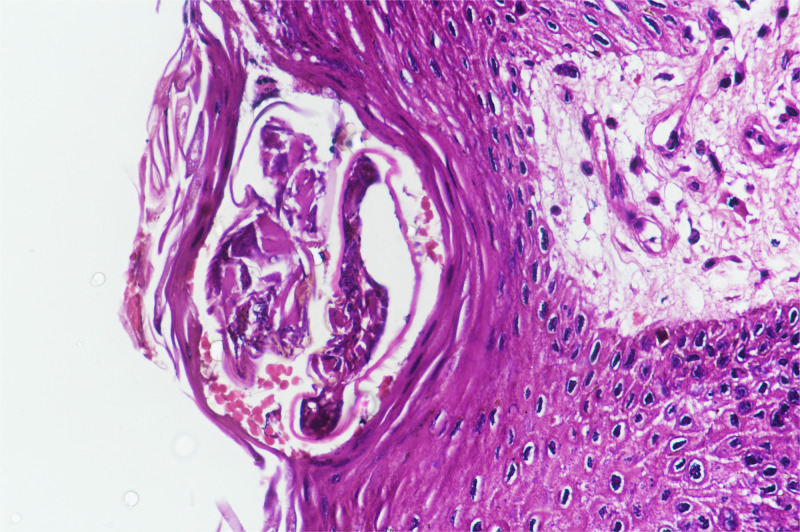
High-power photomicrograph of a skin biopsy showing psoriasiform hyperplasia of the epidermis, with the stratum corneum showing parakeratosis and a mite of Norwegian scabies. The underlying papillary dermis shows dilated capillary channels and supra-papillary thinning (hematoxylin–eosin stain, 40× magnification). The epidermal changes are common to both psoriasis and crusted scabies; however, the presence of supra-papillary thinning and dilated capillary channels may point to persistent psoriatic activity in the skin. This figure appears in color at www.ajtmh.org.

**Figure 4.**
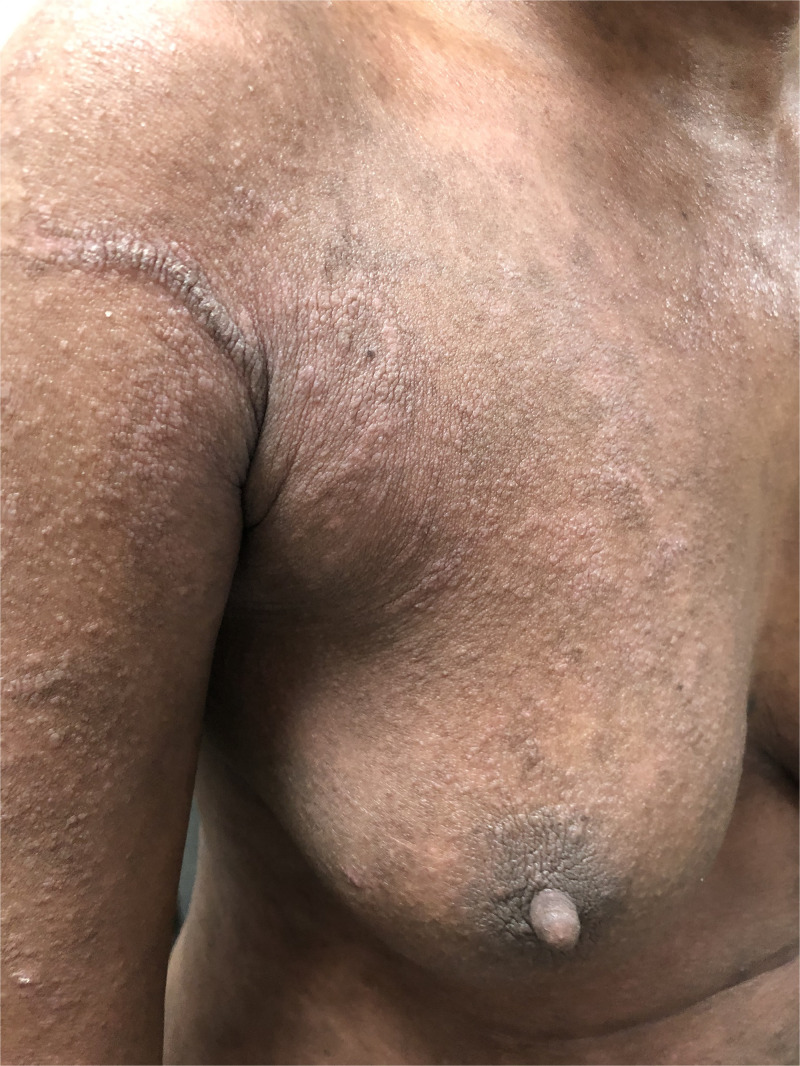
Significant clearing after a single dose of ivermectin. However, a prominent burrow is now visible over the right axillary region, a scraping of which revealed scabies mites, suggesting the need for prolonged treatment. This figure appears in color at www.ajtmh.org.

However, 1 year later, she presented with thick, grayish yellowish crusting, which was extensive over the body (Figure 1), while taking methotrexate 10 mg/week (cumulative methotrexate dose, 630 mg). A mineral oil preparation from the crust demonstrated scabies mites and eggs (Figure 2). Histopathology showed numerous mites within the stratum corneum (Figure 3). A diagnosis of extensive crusted scabies was confirmed. The patient was moved to an isolation room and was put on weekly ivermectin. Remarkable improvement occurred after the first dose (Figure 4), with almost complete resolution of crusting. Repeat doses were given weekly for an additional 3 weeks. The patient has not had any further recurrence of scabies for the past 2 years, although her psoriasis continues to necessitate systemic treatment intermittently.

The older literature is contradictory with reports of both flare of scabies with methotrexate^1^ as well as its effective use for extensive, recalcitrant crusted scabies.^2^ The recurrent development of crusted scabies in our patient while on weekly methotrexate supports the former contention. The association of crusted scabies with methotrexate is not surprising considering the premise that crusted scabies results from a predominant T-helper 1 (Th1) host immune response in contrast to ordinary scabies, in which a T-helper 2 (Th2) response predominates.^3^ Methotrexate is known to suppress the Th1 cellular response, tipping the balance toward Th2 immunity. Crusted scabies has been shown to present with recurrences after long intervals of time (6–12 months), and molecular analysis on sequential populations of mites from such cases have shown the mites to be genetically more similar to each other than mites from other patients, suggesting inadequate treatment—rather than reinfestation—as the cause of relapses.^4^ On the basis of these observations, repeat dosing of ivermectin, three to five times, has been recommended to prevent recurrences of crusted scabies.^4^
